# Image quality robustness of HyperSight‐enabled versus conventional iterative cone‐beam CT across patient size and imaging geometry

**DOI:** 10.1002/acm2.70874

**Published:** 2026-10-20

**Authors:** Nicholas Nelson, Sorour Hosseini, Geoff Nelson, Thomas Martin, Bradley Nordin, Jessica Huang, Hui Zhao

**Affiliations:** ^1^ Department of Radiation Oncology University of Utah Salt Lake City Utah USA

**Keywords:** adaptive radiotherapy, cone‐beam computed tomography (CBCT), contrast‐to‐noise ratio, Hounsfield units, HyperSight, iterative reconstruction, image quality, spatial resolution

## Abstract

**Background:**

Recent advances in cone‐beam computed tomography (CBCT) have led to the development of next‐generation CBCT systems with enhanced image quality and quantitative imaging capabilities. These advances are particularly relevant in the context adaptive radiation therapy, where improved soft tissue visualization and Hounsfield unit (HU) stability are increasingly important for target localization and CBCT‐based dose calculation.

**Purpose:**

To determine how image quality and HU stability change with increasing phantom size, non‐cylindrical geometries, and exposure level, and compare the robustness of Acuros‐based HyperSight‐enabled iterative CBCT (HS‐iCBCT) and conventional iterative CBCT on a TrueBeam (TB‐iCBCT) under these conditions.

**Methods:**

The CatPhan® 604 phantom, combined with custom annular extensions, was used to simulate increasing patient size and non‐cylindrical imaging geometries. Three configurations (no annulus, small annulus, and large annulus) were imaged across a range of exposure levels (mAs) using HS‐iCBCT and TB‐iCBCT. Image evaluation metrics included noise, contrast‐to‐noise ratio (CNR), low‐contrast visibility (LCV), image uniformity, spatial resolution, and Hounsfield unit (HU) constancy.

**Results:**

For the no‐annulus configuration, image quality was comparable between systems, with slightly lower noise observed for TB‐iCBCT. With increasing phantom size through the added annuli, TB‐iCBCT exhibited substantial degradation in noise, contrast, and uniformity, whereas HS‐iCBCT maintained more consistent performance. Spatial resolution was primarily dependent on field of view and was similar between systems, with smaller fields of view provided improved spatial resolution. HU constancy degraded with increasing phantom size and material density for both systems but remained substantially more stable for HS‐iCBCT.

**Conclusions:**

HS‐iCBCT demonstrated improved robustness to patient size and imaging geometry, providing superior image quality and HU stability compared to TB‐iCBCT. These improvements may support potential lower‐dose imaging and more reliable performance in larger patients and non‐cylindrical imaging geometries commonly encountered in clinical radiotherapy.

## 1 INTRODUCTION

Cone‐beam computed tomography (CBCT) has become an essential component of modern image‐guided radiotherapy (IGRT), enabling patient positioning, target localization, and increasingly, adaptive radiation treatments. However, CBCT image quality remains inferior to that of the fan‐beam CT systems used in simulation and diagnostic imaging, particularly with respect to image noise, Hounsfield unit (HU) accuracy, and low‐contrast detectability. These limitations are further exacerbated in clinical scenarios involving larger patients, where increased cone beam‐induced scatter, beam hardening, and photon starvation degrade image quality.^1^, ^2^, ^3^, ^4^

Recent advances in CBCT technology, including iterative reconstruction techniques and hardware improvements, have aimed to address these challenges. The HyperSight‐enabled iterative CBCT (HS‐iCBCT) system represents one such advancement through incorporation of updated detector technology and advanced reconstruction methods designed to reduce cone beam‐induced imaging artifacts and improve HU fidelity.^5^, ^6^, ^7^, ^8^, ^9^, ^10^, ^11^, ^12^, ^13^, ^14^, ^15^, ^16^ On Halcyon systems, the ring‐based gantry design allows for rapid CBCT acquisition (∼6 seconds), a capability currently not achievable on C‐arm‐based TrueBeam systems, resulting in reduced motion artifacts and improved workflow efficiency. In contrast, conventional iterative CBCT implementations, such as those widely available on the TrueBeam platform (TB‐iCBCT), remain widely used for image guidance but may exhibit performance degradation under more challenging imaging conditions, particularly for large patients and non‐cylindrical patient geometries that produce asymmetric attenuation and increase scatter relative to idealized cylindrical phantoms. Accordingly, this study evaluates the overall clinical performance of two commercially available CBCT implementations that differ in detector technology, reconstruction approach, and imaging platform.

A comprehensive evaluation of CBCT image quality should include not only standard cylindrical phantoms, but also more clinically realistic geometries that better approximate patient anatomy.^5^ Variations in patient size and shape can influence image quality metrics such as noise, contrast, and HU constancy. Despite this, many prior studies have evaluated CBCT image quality under controlled conditions using standardized phantoms and acquisition protocols, which may not fully capture the variability encountered in clinical imaging scenarios.^2^, ^4^, ^17^

While many CBCT image quality studies have focused on standard cylindrical phantoms, this study evaluates image quality across increasing phantom size, non‐cylindrical geometries, and a range of exposure levels to better reflect clinical imaging conditions. The CatPhan® 604 phantom, combined with a set of custom‐designed annular extensions, was used to simulate increasing patient thickness and non‐cylindrical geometries. Image quality was evaluated across a range of exposures using metrics including noise, contrast‐to‐noise ratio (CNR), low‐contrast visibility (LCV), and spatial resolution. In addition, HU constancy was assessed across the range of exposures and geometries—which may carry implications for adaptive radiation therapy applications. This approach enables evaluation of performance differences between the two imaging systems under conditions representative of clinical radiotherapy imaging.

## 2 METHODS

### 2.1 Image quality phantom and geometries

The CatPhan® 604 (The Phantom Laboratory, Inc., Greenwich, NY) was used to evaluate image quality on both HyperSight and TrueBeam iCBCT platforms. CatPhan® 604 is a 20 cm diameter cylindrical phantom consisting of three distinct modules for image quality evaluation: CTP732, CTP730, and CTP729. The first module, CTP732, contains nine known‐material inserts for CT‐number or HU sensitometry, a set of line pairs ranging from 1 to 15‐line pairs per centimeter (lp/cm) for spatial resolution evaluation, and a set of tungsten beads and wires for measurement of the modulation transfer function (MTF). The known‐material inserts consist of air, polymethyl pentene (PMP), low‐density polyethylene (LDPE), polystyrene, acrylic, bone 20%, Delrin, bone 50%, and Teflon. The second module, CTP730, is a low‐contrast module that consists of 40 mm long rods with varying diameters and varying contrast levels. The last module, CTP729, is a module that is cast from uniform materials to be within 2% of water's density that allows for noise and uniformity assessment within a homogenous region of tissue.

Three unique geometries were evaluated to characterize image quality as a function of patient thickness through the introduction of two different custom‐made phantom annuli, hereby referred to as the “small” and “large” annuli. The annuli were machined in‐house out of acrylic and designed to represent a patient laying in a supine position on the treatment couch—a commonly employed treatment position in radiotherapy—which differs from the CatPhan's perfectly cylindrical geometry. The inclusion of the small annulus yielded a maximum thickness of 32 cm while the large annulus yielded a maximum thickness of 40 cm. A summary of the geometrical configurations of the CatPhan® 604 and added annuli are shown in Figure 1.

**FIGURE 1:**
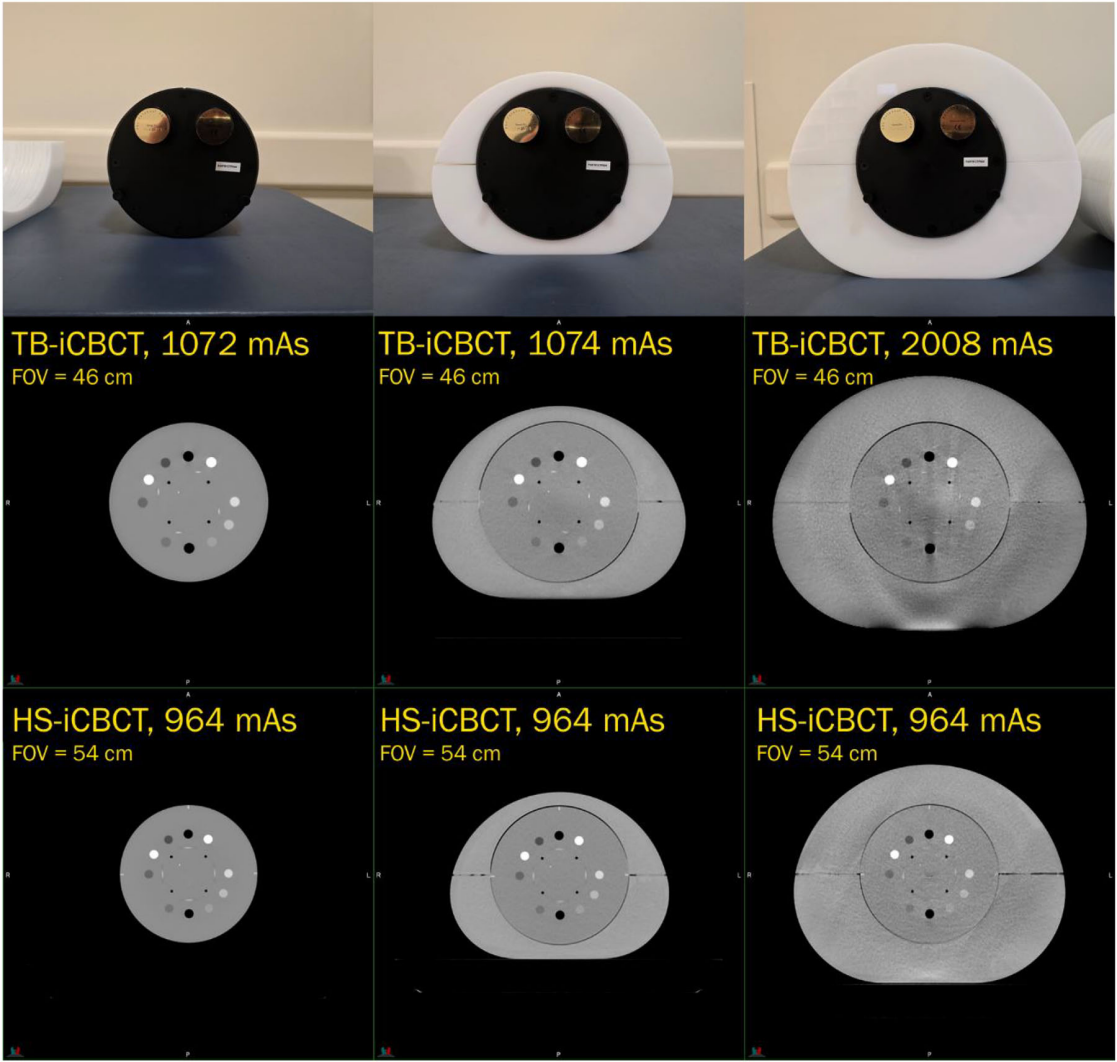
Photographs (top row) and axial CBCT slices (middle and bottom rows) containing the three phantom geometries evaluated: no annulus (left), small annulus (middle), and the large annulus (right). The middle row contains TB‐iCBCT scan results while the bottom row displays the HS‐iCBCT scans. For the HS‐iCBCT scans, a FOV of 54 cm is displayed while the FOV for the TB‐iCBCT scans was 46 cm.

### 2.2 Imaging systems and image acquisition

CBCT images were acquired on both the Halcyon and TrueBeam platforms. The Halcyon MR4.0 system was equipped with HyperSight hardware, whereas the TrueBeam utilized its conventional kV imaging system. Both systems utilized amorphous silicon (a‐Si) flat‐panel detectors with CsI scintillators; however, the HyperSight detector has a larger active area (86 × 43 cm^2^) and incorporates a 15:1 focused anti‐scatter grid, compared with the 40 × 30 cm^2^ detector and 10:1 anti‐scatter grid of the conventional TrueBeam imaging system.^15^, ^16^ Images acquired with HyperSight were reconstructed using the HS‐iCBCT Acuros reconstruction algorithm, whereas TrueBeam images were reconstructed using conventional iterative reconstruction. HS‐iCBCT Acuros incorporates Acuros CTS (computer tomography scatter), a model‐based scatter correction approach that deterministically solves the linear Boltzmann transport equation to estimate photon scatter within the imaged object. Acuros CTS uses an initial reconstruction to generate a material and density map of the imaged object and models photon transport through the object and imaging system, including the anti‐scatter grid and detector response. The resulting scatter estimate is then used to correct the projection data prior to a final image reconstruction.^12^, ^13^ Thus, although both systems employ iterative CBCT reconstruction, the implementations evaluated in this study differ in detector characteristics, acquisition geometry, and reconstruction methodology.

For TB‐iCBCT, a fixed field of view (FOV) of 46 cm was utilized, whereas HS‐iCBCT images were reconstructed using 36 and 54 cm FOVs to bracket the TB‐iCBCT FOV. The three phantom geometries described in Section 2.A were then scanned under various levels of exposure (mAs). All HS‐iCBCT scans utilized exposures that ranged from 132 to 964 mAs for all phantom geometries evaluated. For TB‐iCBCT, exposures of 134 to 1074 mAs were evaluated for the no annulus and small annulus phantom geometries; for the large annulus phantom on TB‐iCBCT, exposures of 268 to 2008 mAs were required to compensate for the increased phantom thickness. This increase in mAs was neither required nor performed for HS‐iCBCT to yield CBCT images sufficient for image quality characterization. Both imaging platforms utilized a tube potential of 125 kVp for image acquisition.

### 2.3 Image quality metrics

The image quality metrics evaluated in this work consist of image noise, CNR, LCV, image uniformity, and spatial resolution. In addition to these image quality metrics, HU constancy was also evaluated with respect to exposure and phantom size. To accomplish this, quantitative analysis was performed using specific regions of interest (ROI) to calculate image quality metrics specific to each CBCT scan. MIM (MIM Software, Beachwood, OH) was utilized to volumetrically contour the cylindrical material inserts in the insert region and noise evaluation regions in the noise region of CatPhan® 604. Figure 2 contains axial slices insert and noise regions of the CatPhan® 604, along with corresponding ROIs that were used to quantify the image quality metrics evaluated in this work. Within the insert region, all material inserts were contoured using 0.9 cm diameter ROIs, along with four background ROIs of 3 cm diameter. All cylindrical ROIs had a length of 1.8 cm, corresponding to volumes of 1.15 and 12.7 cc for insert and background regions, respectively. Noise evaluation was performed in accordance with the recommendations of AAPM TG‐233, which consists of five ROIs with a diameter of 1% of phantom area placed at the center and the 12, 3, 6, and 9 o'clock positions.^18^ The diameters of each ROI were 2 cm for noise and uniformity evaluation, and peripheral ROIs placed one diameter away from the phantom edge.

**FIGURE 2:**
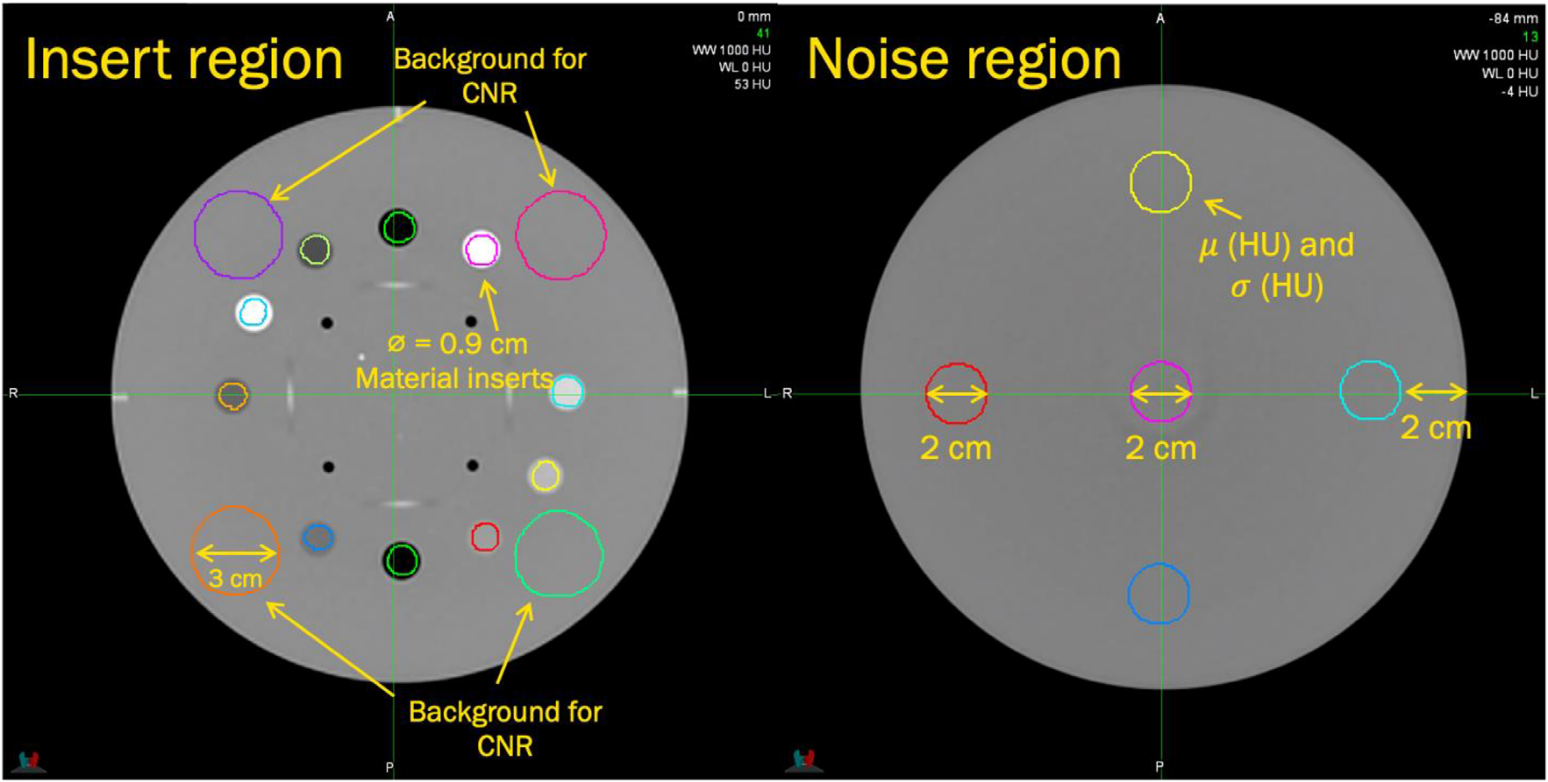
Axial slices of CatPhan® 604 insert region (left) and noise assessment region (right) and corresponding ROIs that were used for image quality quantification. In the insert region, four background ROIs of 3 cm diameter each were utilized in addition to 0.9 cm diameter insert‐specific ROIs. Noise region ROIs consisted of onecentrally located ROI and four peripheral ROIs, each 2 cm in diameter.

#### 2.3.1 Noise

Image noise is defined as the standard deviation of pixel values within some homogenous ROI. Image noise was calculated using the five ROIs shown in Figure 2.^18^ Due to presence of image non‐uniformity across the five ROIs, noise was calculated and reported as a percentage of the mean pixel value for all TB‐iCBCT and HS‐iCBCT scans as a function of image exposure, or mAs, using the following equation,

$$\mathrm{Noise}\left(\%\right)=100\%\cdot \frac{1}{N}\sum_{N=1}^{N=5}\left(\frac{\sigma_{N}}{\mu_{N}+1000}\right)\langle /ieqn\rangle ,$$

where σ and μ are the standard deviation and mean pixel values in each noise ROI, expressed in HU. Due to μ values falling close to zero in the denominator, 1000 HU was added to all μ values.

#### 2.3.2 Contrast

Image contrast was evaluated through calculation of the CNR, which compares the signal difference between two regions scaled to the noise using the following equation by Stützel et al.^19^:

$$CNR=\frac{\left|\mu_{Insert}-\mu_{BG}\right|}{\sqrt{\sigma_{Insert}^{2}+\sigma_{BG}^{2}}}$$

where $\mu_{Insert}$ and $\mu_{BG}$ are the mean CT numbers for the material insert ROI and the background ROIs, and $\sigma_{Insert}$ and $\sigma_{BG}$ are the standard deviations of the mean values in each respective ROI. CNR provides insight into the contrast of one material type relative to the image background, which is typically tissue‐equivalent media. Another metric, the LCV refers to the ability to distinguish between materials with similar attenuation properties. The method of Lehmann et al.^20^ was employed in this work to quantify distinguishability between the polystyrene and LDPE inserts using the equation below,

$$LCV=2.75\times \frac{\sigma_{Poly}+\sigma_{LDPE}}{\mu_{Poly}-\mu_{LDPE}}$$

where $\sigma_{Poly}$ and $\sigma_{LDPE}$ are the standard deviations of the HU values in the polystyrene and LDPE inserts and $\mu_{Poly}$ and $\mu_{LDPE}$ are the mean HU values in the insert ROIs. Lower LCV values indicate an increased ability to distinguish between the two materials while higher CNR values represent an improved contrast of a given material relative to background.

#### 2.3.3 Image uniformity

Image uniformity was evaluated by quantification of the uniformity index (UI), which describes the amount of “cupping” or “capping” present in an image, using the equation below,

$$UI=\mu_{Periphery}\;-\mu_{Center}$$

where $\mu_{Center}$ and $\mu_{Periphery}$ are the average HU measure in the central ROI and four peripheral ROIs in Figure 2, respectively. In this work, UI carries units of HU with an ideal UI yielding zero, while a UI greater than zero would indicate a cupping artifact while a UI less than zero would indicate a capping artifact.

#### 2.3.4 Spatial resolution

Spatial resolution was evaluated by computing the relative modulation transfer function, or rMTF, for the bar patterns in CatPhan® 604 using pylinac, an open‐source python‐based image analysis software.^21^ For each bar pattern present in the CTP732 module, Pylinac computes the modulation of each pattern using the equation below,

$$\;M_{lp/mm}=\frac{I_{max}-I_{min}}{I_{max}+I_{min}}$$

where $I_{max}$ and $I_{min}$ are the maximum and minimum HU values measured across a line profile ROI over a given bar pattern. From there, a relative modulation transfer function (rMTF) was computed by normalizing the modulation of each bar pattern to that of the lowest‐frequency bar pattern (1 lp/cm), as function of spatial frequency in lp/cm.

### 2.4 Hounsfield unit (HU) constancy

HU constancy, or stability, was evaluated for both imaging platforms across all geometrical configurations and exposure ranges evaluated. Constancy was evaluated by comparing the maximum deviation between mean HU values in each ROI across the range of exposures evaluated. For HS‐iCBCT, exposures of 132 to 964 mAs were evaluated for all geometrical configurations. For TB‐iCBCT, exposures of 134 to 1074 mAs were evaluated for the no annulus and small annulus cases while the larger annulus case utilized a higher range of exposures of 268 to 2008 mAs.

## 3 RESULTS

### 3.1 Noise

Figure 3 contains plots of the measured image noise as a percentage of the background signal for HS‐ and TB‐iCBCT imaging platforms for the geometrical configurations shown in Figure 1. A fixed field of view (FOV) of 46 cm was utilized for the TB‐iCBCT scans while two FOV's greater and smaller than the TB‐iCBCT FOV was evaluated to provide insight into noise changes with FOV. Image noise, in general, decreased with increasing mAs across all scenarios evaluated as expected. For the no annulus configuration, noise was very similar among both imaging systems for the same exposures, with a slightly reduced noise measured on the TB‐iCBCT images, especially for higher exposures. For the small and large annuli cases, image noise in TB‐iCBCT images started to greatly increase relative to HS‐iCBCT, especially for low exposure settings. For the small annulus, increases in image noise of roughly 2% were observed in TB‐iCBCT relative to HS‐iCBCT at an exposure of 200 mAs. Above roughly 350 mAs, noise differences were within 1%. For the large annulus, the TB‐iCBCT noise was roughly 10% higher than HS‐iCBCT at 350 mAs. At the maximum exposures evaluated for HS‐iCBCT (964 mAs), TB‐iCBCT (2008 mAs) image noise was 2–3% higher than HS‐iCBCT.

**FIGURE 3:**
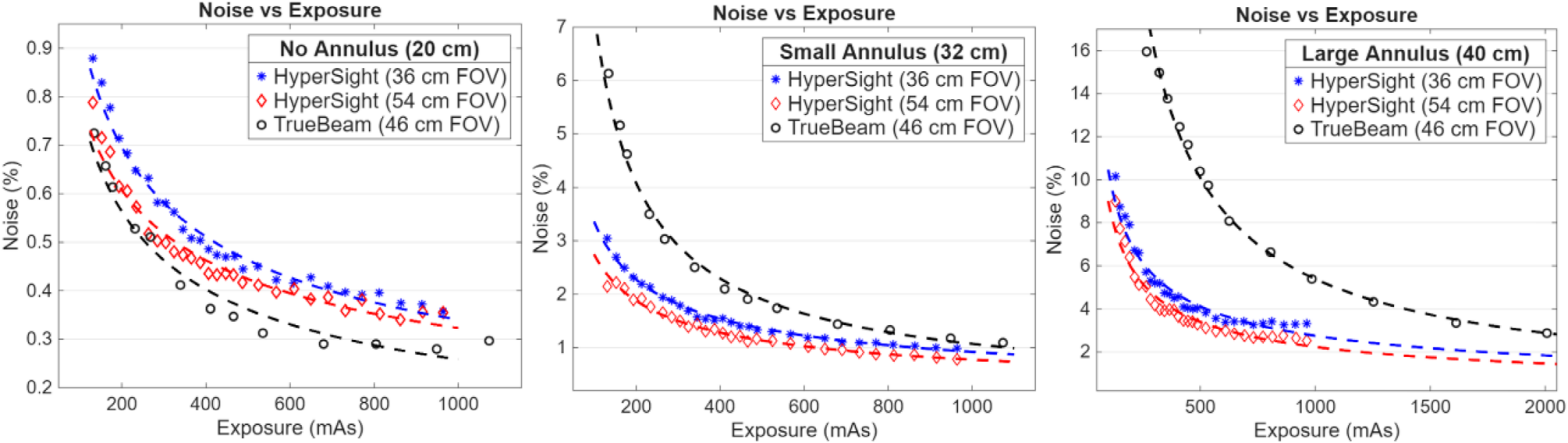
Image noise as a function of exposure (mAs) for the no annulus (left), small annulus (middle), and large annulus (right) cases for TB‐iCBCT (black) with a 46 cm FOV and HS‐iCBCT with 36 (blue) and 54 cm (red) FOVs.

### 3.2 Contrast

Figure 4 contains the CNR as a function of the average measured HU for each insert at a fixed exposure of 964 mAs across each geometrical configuration (no annulus, small annulus, large annulus) and field of view evaluated for HS‐ and TB‐iCBCT scans. In general, CNR was maximized on HS‐iCBCT with the 54 cm FOV relative to TB‐iCBCT for the small and large annuli cases. For the no annulus case, differences in measured CNR were negligible.

**FIGURE 4:**
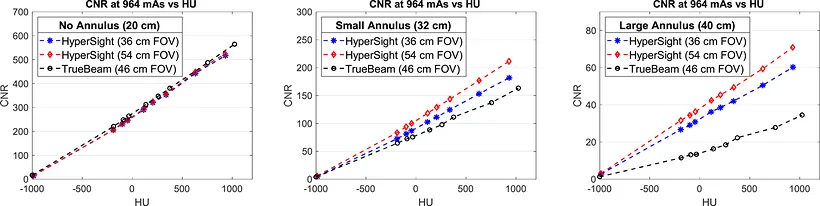
The CNR as a function of HU reported at 964 mAs for the no annulus (left), small annulus (middle), and large annulus (right) cases for TB‐iCBCT (black) with a 46 cm FOV and HS‐iCBCT with 36 (blue) and 54 cm (red) FOVs.

Figure 5 contains the calculated LCV for HS‐ and TB‐iCBCT between polystyrene and LDPE as a function of exposure for all geometric configurations evaluated. For the no annulus case, TB‐iCBCT provided superior LCV relative to HS‐iCBCT, with smaller values of LCV across a majority of the exposure range. Similarly to noise and CNR, the introduction of the small and large annuli significantly reduced LCV for TB‐iCBCT relative to HS‐iCBCT. For HS‐iCBCT, LCV was found to be insensitive to changes in FOV.

**FIGURE 5:**
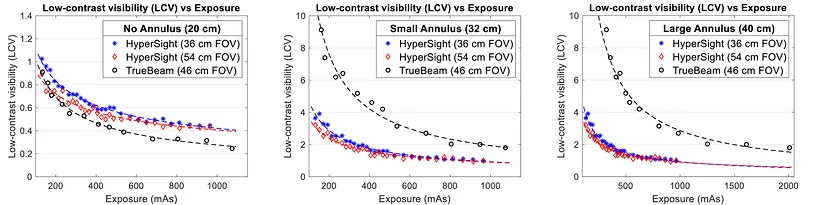
The LCV as a function of exposure (mAs) for the no annulus (left), small annulus (middle), and large annulus (right) cases for TB‐iCBCT (black) with a 46 cm FOV and HS‐iCBCT with 36 (blue) and 54 cm (red) FOVs.

### 3.3 Image uniformity

Figure 6 contains the image uniformity results for HS‐ and TB‐iCBCT for the three geometrical configurations and range of exposure values. HS‐iCBCT demonstrated superior uniformity compared to TB‐iCBCT for the no annulus and small annulus case across the entire exposure range. For the large annulus case, uniformity between HS‐ and TB‐iCBCT were comparable and rather poor (UI > ±10) for lower ranges of exposures below 500 mAs. Between 500 and roughly 900 mAs, HS‐iCBCT outperformed TB‐iCBCT, with UIs ranging from 10 to ‐10, passing through 0 at around 700 mAs. Above 900 mAs, HS‐ and TB‐iCBCT uniformity were comparable. The maximum exposure for HS‐iCBCT evaluated in this work was 964 mAs. Between the two FOVs evaluated for HS‐iCBCT, a slight uniformity improvement was observed for the larger 54 cm FOV, a result that can be likely attributed to increased voxel size and volume averaging.

**FIGURE 6:**
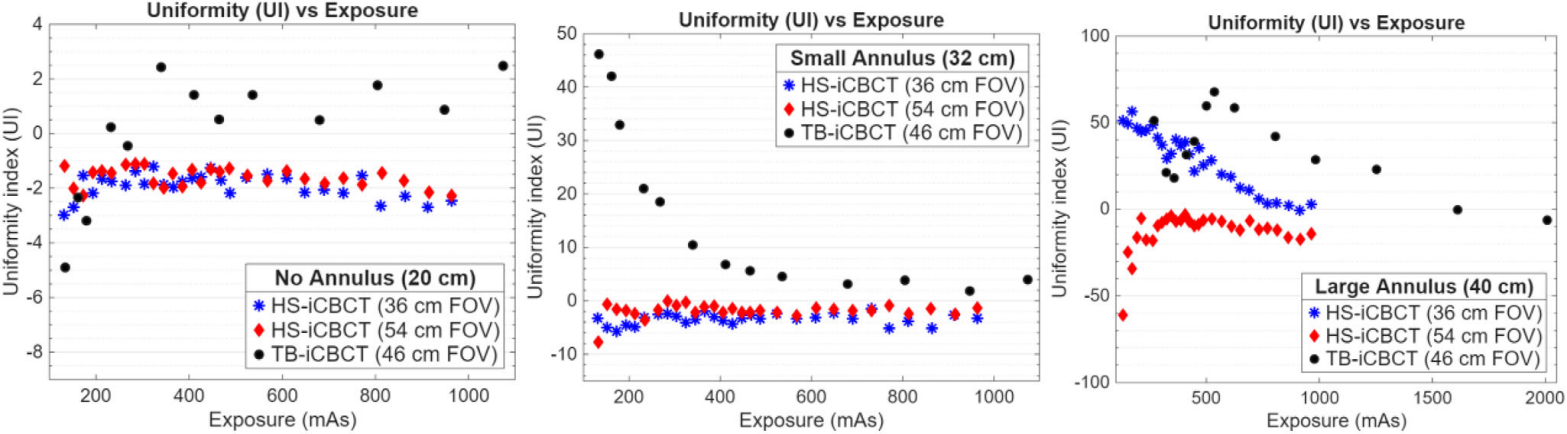
Uniformity index (UI) plotted as a function of exposure (mAs) for the no annulus (left), small annulus (middle), and large annulus (right) cases for TB‐iCBCT (black) with a 46 cm FOV and HS‐iCBCT with 36 (blue) and 54 cm (red) FOVs.

### 3.4 Spatial resolution

Figure 7 contains the computed relative MTF (rMTF) for TB‐iCBCT and HS‐iCBCT for each of the geometrical configuration evaluated in this work. Overall, the rMTF was largely dominated by the FOV as shown by the difference in rMTF between the 36 and 54 cm FOVs evaluated for HS‐iCBCT. Spatial resolution was also found to be relatively independent of phantom size, where changes in the rMTF_10%_ across the three phantom geometries were at or within 0.3 lp/cm of each other. For the no annulus case, the rMTF_10%_ for HS‐iCBCT was 5.5 and 4.3 lp/cm for the 36 and 54 cm FOVs. For the TB‐iCBCT no annulus case, the rMTF_10%_ was measured to be 4.5 lp/cm.

**FIGURE 7:**
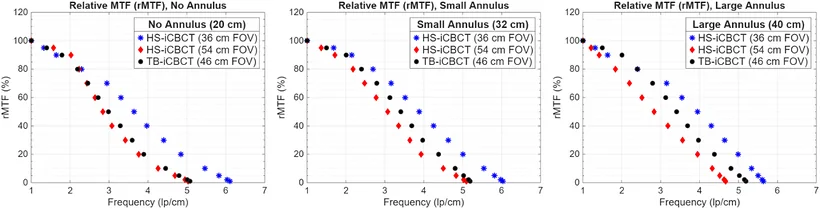
Relative MTF (rMTF) plotted as a function of frequency in line‐pairs per centimeter (lp/cm) for the no annulus (left), small annulus (middle), and large annulus (right) cases for TB‐iCBCT (black) with a 46 cm FOV and HS‐iCBCT with 36 (blue) and 54 cm (red) FOVs.

Considering that TB‐iCBCT utilized a fixed reconstruction FOV of 46 cm, a direct comparison with HS‐iCBCT with a 46 cm FOV was not performed. However, these results suggest that spatial resolution is primarily governed by FOV, with minimal differences between HS‐iCBCT and TB‐iCBCT.

### 3.5 HU constancy

Figure 8 contains the HU constancy as a function of exposure for the PMP, acrylic, and 50% bone material inserts of the CatPhan® 604 for both TB‐iCBCT and HS‐iCBCT across all geometrical configurations. These material inserts were selected to represent a range of clinically relevant densities. The manufacturer‐reported HU ranges for the CatPhan® 604 inserts are based on 120 kVp acquisitions, whereas all CBCT data in this study was acquired at 125 kVp. As a result, small systematic offsets in the measured HU relative the published ranges may be expected.

**FIGURE 8:**
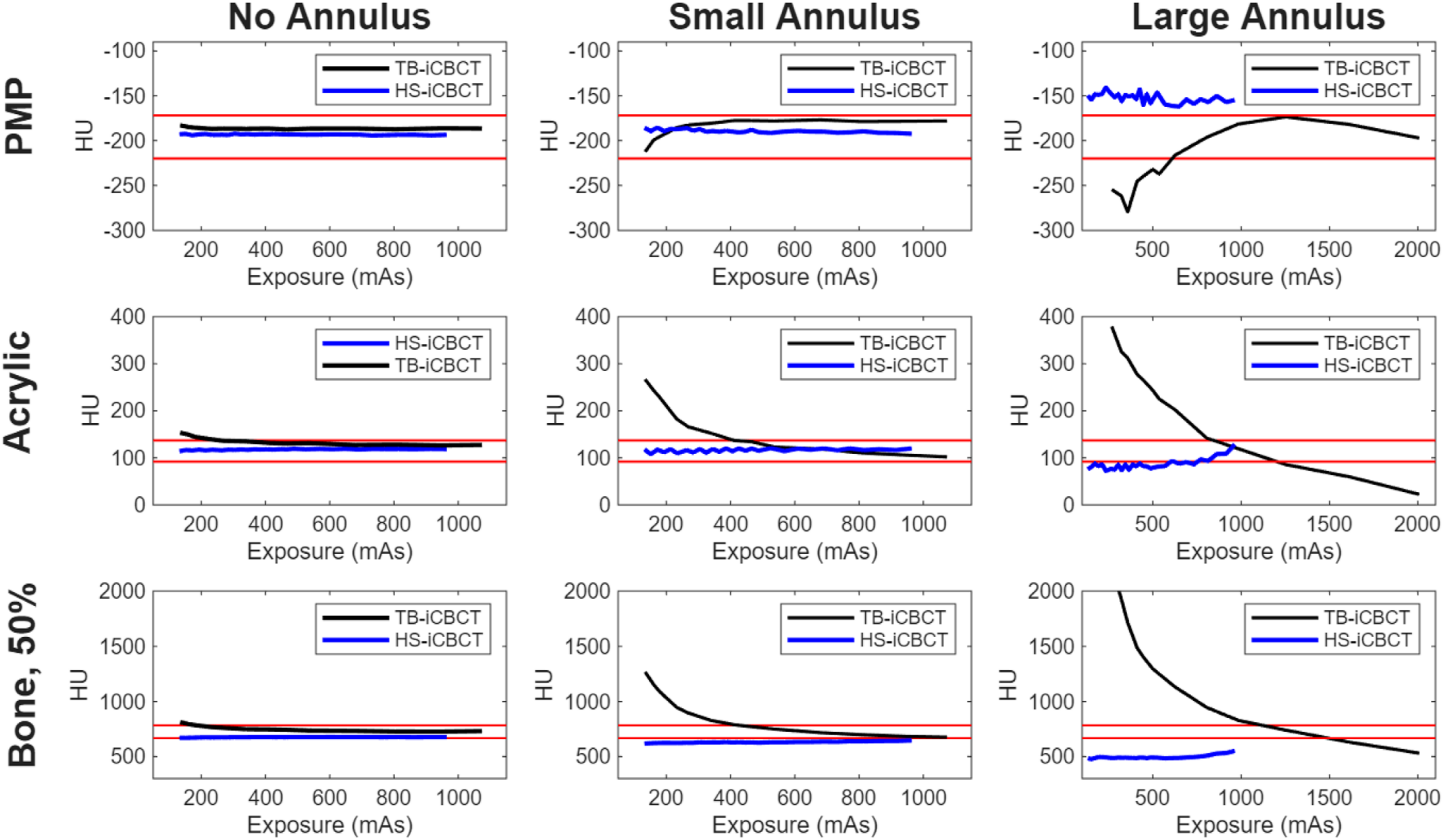
Plots of HU values as a function of exposure (mAs) for TB‐iCBCT (black) and HS‐iCBCT (blue) for PMP (top), acrylic (middle), and 50% bone (bottom) CatPhan® 604 inserts for the no annulus (left), small annulus (middle) and large annulus (right) configurations. Red lines indicate the manufacturer‐reported HU range for each material (120 kVp reference).

Overall, HS‐iCBCT demonstrated improved HU constancy compared to TB‐iCBCT across all configurations and material inserts. For the no‐annulus case, HS‐iCBCT measurements remained within the expected HU range for all materials, while TB‐iCBCT deviated at low exposures (< 200 mAs) for acrylic and bone. In the small annulus configuration, TB‐iCBCT remained outside the expected range for acrylic and bone at exposures below ∼400 mAs, whereas HS‐iCBCT remained largely stable, with a modest HU offset (∼50 HU) observed for bone.

For the large annulus configuration, HU constancy was reduced for both systems, with substantially larger variability observed for TB‐iCBCT. TB‐iCBCT exhibited a strong dependence on exposure, particularly for acrylic and bone, with only a narrow mAs range yielding values within the expected HU range. In contrast, HS‐iCBCT demonstrated reduced variability with exposure but exhibited systematic HU offsets, including ∼20 HU for PMP and up to 100–150 HU for bone.

These results indicate that while HS‐iCBCT provides improved HU stability, deviations in the measured HU persist under large, non‐cylindrical geometries. For such scenarios, particularly in larger patients, the use of geometry‐ or protocol‐specific HU‐to‐density calibrations may be warranted for accurate dose calculation.

## 4 DISCUSSION

The exposure levels investigated in this study closely reflect those used in standard clinical CBCT protocols. Head imaging is typically performed at approximately 150–300 mAs, consistent with the no‐annulus configuration, while pelvis imaging commonly employs exposures near 1000–1500 mAs, comparable to the small annulus case. For larger patients, large pelvic imaging protocols often require 1500–1800 mAs, aligning with the large annulus geometry.

Across all image quality metrics evaluated in this work, a clear dependence on phantom size was observed. For the no‐annulus configuration, TB‐iCBCT demonstrated comparable—and in some cases slightly superior—performance relative to HS‐iCBCT, particularly in terms of noise, CNR, and LCV. However, with the introduction of the small and large annuli, HS‐iCBCT demonstrated improved performance across all evaluated image quality metrics. These findings suggest that while both systems perform similarly under ideal imaging conditions (e.g., cylindrical geometry for no‐annulus case), HS‐iCBCT provided increased robustness in the presence of greater attenuation and non‐cylindrical imaging geometries. Importantly, the purpose of this comparison was not to re‐establish the known limitations of conventional TB‐iCBCT, but to characterize how image quality and HU stability change with patient size, imaging geometry, and exposure and to determine whether these effects are mitigated by a newer CBCT implementation.

The degradation in TB‐iCBCT image quality with increasing phantom size is consistent with the expected effects of increased scatter, beam hardening, and reduced photon fluence associated with larger imaging geometries. In contrast, the relative preservation of HS‐iCBCT image quality under these conditions suggests that the combined hardware, acquisition geometry, and reconstruction characteristics of the HS‐iCBCT implementation provide greater robustness under increased attenuation and geometric asymmetry. The ability of HS‐iCBCT to achieve comparable or improved image quality at lower exposures also suggests potential for lower‐exposure imaging relative to TB‐iCBCT, although the magnitude of any achievable dose reduction would depend on the clinical imaging task and acceptable image‐quality thresholds.

HU constancy decreased with increasing phantom size and material insert density for both systems, with a more pronounced effect observed for TB‐iCBCT. This behavior is likely driven by increased scatter and beam energy spectra hardening, which disproportionately affect higher‐density materials and larger imaging geometries. Consistent with the results shown in Figure 8, TB‐iCBCT exhibited substantial exposure dependence and large HU variability for acrylic and bone in the annulus configurations, whereas HS‐iCBCT demonstrated improved stability with reduced sensitivity to mAs with systematic HU offsets for materials in the large annulus case.

The improved HU stability observed with HS‐iCBCT has important implications for quantitative applications such as dose calculation in adaptive radiotherapy. Prior work by Davis *et al.* has shown that HU variations of ±20 HU in soft tissue and ±50 HU in lung and bone typically correspond to dose calculation differences of < 1% in photon radiotherapy if not accounted for; however, the dosimetric impact of HU variations depends on factors including tissue type, patient geometry, treatment energy, dose calculation algorithm, and the treatment plan itself.^22^ In this context, the magnitude of HU variation observed with HS‐iCBCT—particularly for low‐ and medium‐density materials—likely remains within clinically lower acceptable limits.^22^, ^23^, ^24^, ^25^, ^26^ However, the large deviations in HU observed for high‐density material under extreme geometrical conditions suggest that geometry‐ or protocol‐specific HU‐to‐density calibration may still be warranted for accurate dose calculation in these scenarios. Given the growing interest and deployment of CBCT‐based adaptive radiotherapy,^27^ future work should evaluate the dosimetric sensitivity to the HU variability and systematic offsets observed under large patient geometries.

## 5 CONCLUSIONS

HS‐iCBCT demonstrated improved and robust image quality compared to TB‐iCBCT, particularly with increasing phantom size and non‐cylindrical geometries. While performance was comparable under ideal conditions, TB‐iCBCT degraded substantially with increasing thickness, whereas HS‐iCBCT maintained lower noise, improved contrast, and superior HU constancy.

## AUTHOR CONTRIBUTIONS

The author contributions to this article are as follows: study design and conception: N. Nelson and H. Zhao; data collection: N. Nelson, H. Zhao, and S. Hosseini; data analysis and interpretation: N. Nelson, B. Nordin, H. Zhao, G. Nelson, T. Martin, J. Huang; manuscript drafting and preparation: N. Nelson, H. Zhao, G. Nelson, T. Martin, J. Huang.

## CONFLICT OF INTEREST STATEMENT

The authors have no conflicts of interest.

## ETHICAL STATEMENT

Not applicable. No patient data was collected for this study.

## Data Availability

The data that support the findings of this study are available from the corresponding author upon reasonable request.
